# Risk Factors for HIV Infection among Young Thai Men during 2005–2009

**DOI:** 10.1371/journal.pone.0136555

**Published:** 2015-08-26

**Authors:** Ram Rangsin, Khunakorn Kana, Thippawan Chuenchitra, Akachai Sunantarod, Mathirut Mungthin, Supanee Meesiri, Wirote Areekul, Kenrad E. Nelson

**Affiliations:** 1 Department of Military and Community Medicine, Phramongkutklao College of Medicine, Bangkok, Thailand; 2 Armed Forces Research Institute of Medical Sciences (AFRIMS), Bangkok, Thailand; 3 The Royal Thai Army Institute of Pathology (AIP), Bangkok, Thailand; 4 Department of Parasitology, Phramongkutklao College of Medicine Bangkok, Thailand; 5 Department of Epidemiology, Johns Hopkins University Bloomberg School of Public Health, Baltimore, Maryland, United States of America; David Geffen School of Medicine at UCLA, UNITED STATES

## Abstract

**Background:**

Thailand is one of several countries with a continuing generalized HIV epidemic. We evaluated the risk factors for HIV prevalence among 17–29 year old men conscripted by a random process into the Royal Thai Army (RTA) in 8 cohorts from 2005–2009.

**Methods:**

A series of case-cohort studies were conducted among the male RTA conscripts who had been tested for HIV seroprevalence after they were inducted. Men who were HIV positive were compared with a systematic random sample (1 in 30–40) of men from the total population of new conscripts. Each subject completed a detailed risk factor questionnaire.

**Results:**

A total of 240,039 young Thai men were conscripted into the RTA and were screened for HIV seroprevalence between November 2005 and May 2009. Of 1,208 (0.5%) HIV positive cases, 584 (48.3%) men were enrolled into the study. There were 7,396 men who were enrolled as a comparison group. Among conscripts who had an education lower than a college-level, the independent risk factors for HIV infection were age in years (AOR 1.38, 95% CI 1.28–1.48), a history of sex with another man (AOR 3.73, 95% CI 2.70–5.13), HCV infection (AOR 3.89, 95% CI 2.56–5.90), and a history of sex with a female sex worker (FSW) (AOR 1.35, 95% CI 1.10–1.66). Among conscripts who had a college degree, the independent risk factor for HIV infection was a history of sex with another man (AOR 23.04, 95% CI 10.23–51.90). Numbers of sexual partners increased and the age at first sex, as well as the use of condoms for sex with a FSW decreased in successive cohorts.

**Conclusion:**

The HIV seroprevalence among cohorts of 17–29 years old men has remained at about 0.5% overall during 2005–2009. The most significant behavior associated with HIV prevalence was a history of sex with another man. Our data indicate continuing acquisition of HIV among young men in Thailand in recent years, especially among men with a history of same sex behavior.

## Introduction

The HIV epidemic in Thailand has decreased substantially since its peak in the early 1990s. Although Thailand has had considerable success in HIV prevention. It has been estimated that 43,040 new infections will occur during 2012‐2016 [1]. Risk behaviors primarily associated with HIV transmission have changed since the peak of the epidemic. Unlike the HIV epidemics in most developed countries, studies of HIV infection in the Thai population during 1993–1995 found that heterosexual transmission played the major role [2]. Studies during the early phase of the epidemic found that more than 90% of HIV infected men reported having sex with female sex workers, whereas only about 1% had a history of injection drug use (IDU). The peak incidence of the HIV epidemic occurred from 1991 to 1993. Nopkesorn et al [3], Celentano et al [4] and Carr et al [5] reported that the HIV incidence among young Thai military conscripts from the upper northern provinces during the early 1990s were 2.0, 2.5 and 3.2 per 100 person-years, respectively. However, the HIV incidence rate of former military conscripts after discharge from the Royal Thai Army during 1995–1999 was 0.31 per 100 person-years [6]. The decreased incidence among young Thai men, after their discharge from the military was believed to be attributable to the successful national comprehensive HIV prevention efforts, especially the 100% condom campaign [2, 7].

Although Thailand has been successful in decreasing the heterosexual transmission of HIV during commercial sex, recent data suggest a resurgence of incident HIV infections among men who have sex with men (MSM). MSM currently are playing the major role in the current epidemic of HIV infection in Thailand. Recently, van Griensven and his colleagues have documented the significant impact of MSM in maintaining the current HIV epidemic in Thailand from venue-based surveys among MSM in Bangkok. They found that the overall HIV prevalence among MSM increased from 17.3% in 2003 to 28.3% in 2005 and 30.8% in 2007. The estimated annual HIV incidence among young MSM increased from 4.1% in 2003 to 6.4% in 2005, to 7.7% in 2007 [8]. The same group of investigators, in cooperation with the Thai Ministry of Public Health, reported a high prevalence of HIV infection among MSM in other major provinces of Thailand, including Chiang Mai and Phuket where the HIV prevalence rates were 6.9 and 20.0% in 2007, respectively. The HIV prevalence among MSM in two other less cosmopolitan provinces of Udon Thani and Pathalung were 4.7 and 5.5% respectively. The very rapid spread of HIV infection among MSM was also confirmed by a three year follow-up cohort study of MSM in Bangkok from 2006 to 2008 which found that, the overall HIV incidence density was 6.1 per 100 person-years; 6.0 in 2006, 6.3 in 2007 and 5.7 in 2008 [9].

Military conscripts in Thailand comprising men aged 17 to 29 years are a representative national sample of young Thai men [3, 10], because the conscription is conducted using a random lottery selection process which does not excluded men on the basis of a history of homosexuality, illicit drug use, or HIV seropositivity. We studied reported risk behavior among young male Royal Thai Army (RTA) conscripts with HIV compared to a comparison group selected from the same conscription cohort between 2005 and 2009 in order to evaluate the temporal trends of reported behavior associated with HIV infection. In addition, HCV seroprevalence was measured as a surrogate marker for a history of injection drug use (IDU) and other parenteral exposures. These recent data from a representative national sample of young men in Thailand provide very useful epidemiologic data to monitor temporal trends in HIV prevalence and risk behaviors associated with infection since the national HIV prevention program was implemented by the Thai Ministry of Public Health.

## Methods

Men aged 21 years are selected by the RTA for conscription using a lottery system. The lottery is held annually at the district level within each province. Young men register and participate in the lottery in the district of their family residence. Exemptions are available for a small subset of men who are disabled, severely ill, certain religious personnel, some teachers, and a few individuals who participate in alternative military service. Individuals with asymptomatic HIV infection are not excluded from participation in the lottery or subsequent service. Sexual orientation and drug use are not grounds for exemption. Individuals without a recognized exemption who fail to register and participate in the lottery system suffer legal sanctions and economic penalties. Therefore, participation is nearly uniform. Approximately one in ten men who participate in the lottery is randomly chosen in April each year. The total number of participants is about 60,000 new conscripts per year. Induction occurs either in May or November of each year for a two-year duration of military service [11]. Since 2001, the RTA began inducting some volunteers who were aged 17 to 20 or 22 years or older into the military [12]. These men were not selected by the lottery system.

In 1989, the RTA and Thailand Ministry of Public Health began to provide HIV testing for all newly inducted conscripts as part of the national HIV surveillance system. The surveillance system of military conscripts includes serologic testing for HIV and a short questionnaire containing demographic data (without behavior information). The collection of blood samples and self-administered questionnaires were supervised by the personnel from the local military hospital at each base. All blood samples and questionnaires were processed at the Armed Forces Research Institute of Medical Sciences (AFRIMS) and the Royal Thai Army Institute of Pathology (AIP) in Bangkok.

During the first two weeks after induction, in May and November each year, a venous blood sample was collected from each conscript after HIV pretest counseling was provided and an informed consent to participate in the surveillance was obtained. The serum sample was sent to the AIP laboratory in Bangkok for HIV antibody testing (enzyme-linked immunosorbent assay) and each positive sample was confirmed by a Western Blot test using licensed commercial reagents. All serum samples remaining from the HIV testing were stored at -70°C at the AFRIMS laboratory. These processes involve about 30,000 blood samples every 6 months. The Standard Operation Procedure (SOP) for providing the HIV test results under the surveillance system of the RTA included 2 steps. The first step involved the HIV testing for the surveillance purpose. The HIV test results of this first step of HIV testing were usually available in the third month after the conscript induction. HIV positive men identified during this step served as the case group of our current study. The second step involved confirming of the HIV test results for the HIV positives by asking the HIV positive conscripts to provide another blood sample to be retested. This confirmation step was to ensure that the HIV positive test results would be provided to the right person. The final test results were made available for posttest counseling at approximately 4–5 months after the induction. The test results were kept confidential and sent to the designated physicians or trained nurses in the responsible regional military hospitals for posttest counseling. HIV positive conscripts remained in the service unless their health status precluded continuing service. Those who remained in the service were treated for HIV when indicated.

### Study population

The study population consisted of young Thai men aged 17 to 29 years, inducted into the military service each year from November 2005 to May 2009. The total number of young men participating in the national HIV sero-surveillance was 240,039 men during the study period. Cases enrolled in this study were men with an HIV positive ELISA and Western blot test result. The comparison group was selected using a systematic random sampling of 1-in-30 (November 2005, May 2006, November 2008, May 2009), 1-in-35 (November 2006, May 2007, November 2007), and 1-in-40 (May 2008), based on the lists of names of the new conscripts taken from their rosters in each induction round and military base.

### Questionnaire process

During the first two months of the induction, the sampled men (1:30–40) from every training unit nationwide were interviewed before the official HIV test results became available. Because these sampled men were selected systematically, some HIV positive cases with test results reported later were included in this group.

After we obtained the HIV test results of the first step of the surveillance system from the RTA Institute of Pathology, HIV positive men were invited to participate in the study, and interviewed. This enrollment process among the HIV positive men was usually performed during the third month after the conscripts were inducted.

The list of cases and the comparison group (sampled men) was prepared and was grouped according to military unit nationwide. The list of study subjects was sent to a designated health worker, usually a nurse or public health officer, at each of the 37 regional military hospitals to administer the questionnaire. These questionnaires were completed before the HIV test results were reported to the men to avoid information bias. A standardized questionnaire was based on the questionnaire used to identify HIV risk behavior during the previous studies of HIV risk factors among Thai conscripts from 1991 to 1998. The factors of interest included number and types of lifetime sexual partners, sex with female or male sex workers, sex with non-commercial female partners, sex with other men, age at first sexual intercourse, frequency of condom use with each type of partner, history of sexually transmitted infections (STIs), alcohol consumption, blood transfusion, injection drug use and recreational drug use, tattoos medical and non-medical injections, surgery, and hepatitis symptoms.

### Laboratory evaluation

#### HIV serology

HIV antibodies were detected by an enzyme-linked immunosorbent assay (Murex HIVAg/Ab Combination, Murex Biotech Ltd, UK) for screening and reactive specimens were tested in duplicate by Enzygnost Anti-HIV 1/2 Plus assay (Dade Behring Marburg GmbH, Germany) and confirmed using a licensed Western Blot test (Abbott Laboratories).

#### HCV serology

HCV antibodies were detected using a third-generation enzyme immunoassay EIA 3.0 (Murex anti-HCV version 4.0; Abbott, Kyalami, South Africa). All reactive samples were retested in duplicate using the same EIA 3.0 assay. The level of reactivity in the EIA was calculated from two or more of the three test results [13]. The signal to cut-off ratio recommended by the Centers for Disease Control and Prevention (CDC) was used to define a positive HCV EIA test. An anti-HCV positive test was defined as a positive test result from both tests [14].

### Data analysis

Mean and standard deviation were used to describe continuous data. Percentage was used to describe categorical data. The χ^2^ or Fisher’s exact test for categorical variables and the Student’s t test for continuous variables were used in univariate analyses to compare the effects of potential risk factors for HIV infection between the HIV cases and the sampled men comparison group. The odds ratios and 95% confidence intervals of demographic and behavioral variables associated with HIV seroprevalence were evaluated using univariate analysis. A multiple logistic regression model was used to identify the independent effect of potential risk factors. A p-value less than 0.05 was considered statistically significant. The trends of proportions of high risk factors for HIV infection during the study period were analyzed among the sampled men.

### Ethics statement

The study protocol was reviewed and approved by the Institutional Review Board of the RTA Medical Department. Written informed consent was obtained from the participants after they read the information sheet and signed the consent form. HIV voluntary counseling and testing (VCT) was given to the potential study participants by the trained persons. Men with HIV and HCV infected men received post-test HIV and HCV counseling at the military hospitals following the standard of HIV/AIDS and HCV treatment in Thailand.

## Results

A total of 240,039 young Thai men, conscripted into the RTA between November, 2005 and May, 2009, participated in the HIV sero-surveillance and comprised the baseline population for this case-cohort study. Of 1,208 (0.5%) HIV-positive men during this period, 584 (48.3%) were enrolled into the study. Among all male conscripts in the same period, 7,396 were enrolled as the randomly sampled controls, 45 of whom were also HIV-positive. The number of study subjects by round of induction is shown in Table 1. The participants were selected from all 330 RTA basic military training units nationwide.

**Table 1:** Number of participants.

Round of inductions	Total conscripts	Total HIV positives (%)	Enrolled sampled men (%)	Enrolled HIV positives (%)	Total enrolled participants
**November 2005**	29,614	158 (0.53)	998 (3.37)	82 (51.90)	1080
**May 2006**	29,858	160 (0.54)	1128 (3.78)	70 (43.74)	1198
**November 2006**	27,706	125 (0.45)	768 (2.77)	78 (62.40)	846
**May 2007**	30,097	143 (0.48)	808 (2.68)	69 (48.25)	877
**November 2007**	27,919	132 (0.47)	772 (2.77)	67 (50.75)	893
**May 2008**	31,805	175 (0.55)	798 (2.51)	83 (47.43)	881
**November 2008**	31,008	153 (0.49)	1050 (3.39)	55 (35.95)	1105
**May 2009**	32,032	162 (0.51)	1074 (3.35)	80 (49.38)	1154
**Total**	240,039	1208 (0.50)	739 (3.08)	584 (48.34)	7980

Because this study was carried out in eight consecutive rounds of new conscripts who were inducted between November 2005 and May 2009, the data provided an opportunity to evaluate trends in several high risk behaviors among the young Thai male population. We found that the mean number of lifetime sexual partners among the sampled men progressively increased from 5.96 (+/- 8.52) in 2005 to 9.30 (+/-12.15) in 2009 (Table 2). In addition, the age at first sexual intercourse decreased over time from 17.44(±2.00) years in 2005 to 16.42(±2.08) years in 2009. Although the proportion of men who reported having sex with a FSW was stable at about 25% during the study years, the proportion of men who reported consistent condom use with FSWs during the last 6 months decreased from 82.3% in 2005 to 70.2% in 2009 (p = 0.04).

**Table 2:** Risk behaviors of sampled men by years of induction, from 2005–2009.

Characteristic	2005	2006	2007	2008	2009	P-Value ^a^
**Number of lifetime sexual partners**						
Mean (±SD)	5.96 (±8.52)	6.82 (±9.45)	7.62 (±10.43)	7.50 (±10.14)	9.30 (±12.15)	<0.001
Median (Min.-Max.)	3.0 (0–40)	3.0 (0–58)	3.0 (0–100)	3.5 (0–80)	4.0 (0–104)	<0.001
**Age at first sexual intercourse (Yrs)**						
Mean (±SD)	17.44 (±2.00)	17.03 (±1.94)	16.83 (±2.10)	16.78 (±2.04)	16.42 (±2.08)	<0.001
Median (Min.-Max.)	17.00 (10–24)	17.00 (10–23)	17.00 (9–24)	17.00 (10–24)	16.00 (8–25)	<0.001
**History of sexual relations with female sex worker**	208/887 (23.45%)	460/1788 (25.73%)	407/1493 (27.26%)	448/1712 (26.17%)	267/1052 (25.38%)	0.38
**Always use condom with a female sex worker during the last 6 months**	93/113 (82.30%)	169/219 (77.17%)	177/215 (82.33%)	153/205 (74.63%)	66/94 (70.21%)	0.04
**History of same sex behavior**	19/954 (1.99%)	63/1853 (3.40%)	58/1419 (4.09%)	50/1718 (2.91%)	45/1070 (4.21%)	0.07
**Always use condom with male sex partners during the last 6 months**	13/21 (61.90%)	41/61 (67.21%)	34/63 (53.97%)	33/49 (67.35%)	15/31 (48.39%)	0.949
**History of non-injecting drug use**	302/978 (30.88%)	596/1873 (31.82%)	566/1571 (36.03%)	822/1838 (44.72%)	482/1065 (45.26%)	<0.001
**History of Injecting drug use**	32/993 (3.22%)	92/1866 (4.93%)	101/1577 (6.40%)	112/1835 (6.10%)	61/1071 (5.70%)	0.004
**History of HIV testing**	180/978 (18.40%)	312/1861 (16.77%)	293/1552 (18.88%)	302/1823 (16.57%)	196/1072 (18.28%)	0.92

^a^ Chi Square for trend

The overall proportion of men who reported a history of same sex behavior was 3.3% (95%CI, 2.94–3.79). The proportion of men who reported same sex behavior increased from 2.0% in 2005 to 4.2% in 2009. The proportion of men who reported consistent condom use during sex with a male partner (48.4%- 67.3%) was consistently lower than those reporting regular condom use for sex with FSWs (70.2%-80.3%) (Table 2).

The proportion of men who reported a history of non-injection drug use increased from 30.9% in 2005 to 45.3% in 2009. The percentage of men who reported injection drug use ranged from 3.2% in 2005 to 6.4% in 2007. The proportion of men who reported having had an HIV test before induction into the RTA was 18.9% (95% CI 18.0–19.9).

By univariate analysis the HIV positive men were more likely to have had sex with another man, to have more reported sexual contacts with female sex workers, to have had more lifetime sexual partners and to be hepatitis C positive (Table 3). Among HIV positive men 76 of 576 (1.32%) reported a history of having had an STI compared with 365 of 7387 (0.49%) of the comparison group, p <0.001 (Table 3). These STIs included gonococcal and non-gonococcal urethritis, syphilis, condyloma, and penile herpes infections (Table 3). Also the HIV positive men were slightly older, had either only a primary education or a college degree, a higher income, were residents of the upper north or the eastern regions of the country and had a history of injection drug use (Table 3). The HIV prevalence among conscripts from the randomly selected comparison group who reported a lifetime history of having had sex with another man was 3.8%, while 0.5% was found among those who denied ever having sex with another man. The prevalence of HIV infection in those MSM with a college degree was 15.4% while it was only 0.5% in those with a college education who were not MSM.

**Table 3:** Characteristics of study participants and associations with HIV infections.

Characteristic	HIV+ cases (N)	Proportion (%)	Sampled men (N)	Proportion (%)	Crude Odds Ratio	95% CI	P-Value
**Age (Yrs), mean ±SD**		Mean 21.74±1.50		Mean 21.16±1.33	1.3	1.24–1.37	<0.001
17–20	43	7.47	926	12.58	1		
21	261	45.31	5,035	68.43	1.12	0.80–1.55	0.514
22–29	272	47.22	1,397	18.99	4.19	3.01–5.85	<0.001
**Region of residence 2 years prior to induction**							
Upper North	66	11.62	707	9.77	1.54	1.05–2.26	0.026
Lower North	48	8.45	555	7.67	1.43	0.95–2.16	0.089
North-East	189	33.27	2,469	34.11	1.27	0.92–1.75	0.155
Central	64	11.27	939	12.97	1.13	0.77–1.65	0.542
East	38	6.69	394	5.44	1.59	1.03–2.48	0.038
Bangkok	114	20.07	1,364	18.84	1.38	0.98–1.95	0.067
South	49	8.63	810	11.19	1		
**Marital status**							
Married	145	25.22	2,077	28.39	1		
Single	430	74.78	5,240	71.61	1.18	0.97–1.43	0.104
**Education**							
Secondary-some college	363	62.48	5112	69.32	1		
Primary	160	27.54	1686	22.86	1.34	1.10–1.62	0.003
College degree	58	9.98	577	7.82	1.42	1.06–1.89	0.019
**Monthly income**							
< US$333.33	380	83.52	4862	89.19	1		
> = US$333.33	75	16.48	589	10.81	1.63	1.25–2.12	<0.001
**History of injecting illicit drug use**							
Never	535	92.56	6,964	94.59	1		
Ever	43	7.44	398	5.41	1.41	1.01–1.95	0.041
**History of non-injecting illicit drug use**							
Never	335	58.26	4,557	62.21	1		
Ever	240	41.74	2,768	37.79	1.18	0.99–1.40	0.061
**Sexual experience**							
None	37	6.7	816	11.35	1		
Exclusive sex with women	432	78.26	6,147	85.48	1.55	1.10–2.19	0.012
Bisexual	50	9.06	192	2.67	5.74	3.65–9.04	<0.001
Exclusive sex with men	33	5.98	36	0.5	20.22	11.37–35.96	<0.001
**Sex with female sex workers**							
Never	352	65.79	5,142	74.18	1		
Ever	183	34.21	1,790	25.82	1.49	1.24–1.80	<0.001
**Condom use with female sex worker last 6 m**.							
No sex	453	83.43	6169	87.94	1		
Always	64	11.80	658	9.38	1.33	1.01–1.74	0.044
Not always	26	4.79	188	2.68	1.88	1.24–2.87	0.003
**Condom use with male partners**							
No sex	503	94.54	6782	98.70	1		
Always	14	2.63	45	0.65	4.20	2.29–7.69	<0.001
Not always	15	2.82	45	0.65	4.49	2.49–8.12	<0.001
**Number of lifetime sexual partners**	Median 5 (0–50)	Median 3 (0–104)			1.02	1.012–1.028	<0.001
0–3	164	37.10	3,255	51.45	1		
> = 4	278	62.90	3,072	48.55	1.8	1.47–2.19	<0.001
**Hepatitis C antibody**							
Negative	542	92.81	7,264	98.22	1		
Positive	42	7.19	132	1.78	4.26	2.98–6.10	<0.001
**History of GC urethritis**							
Never	577	98.80	7380	99.78	1		
Ever	7	1.20	16	0.22	5.6	2.29–13.66	0.001
**History of NGC urethritis**							
Never	531	92.19	7194	97.39	1		
Ever	45	7.81	193	2.61	3.16	2.26–4.42	<0.001
**History of Syphilis infection**							
Never	570	98.96	7365	99.69	1		
Ever	6	1.04	23	0.31	3.37	1.37–8.31	0.008
**History of penile Condyloma**							
Never	559	97.05	7315	99.03	1		
Ever	17	2.95	72	0.97	3.09	1.81–5.28	<0.001
**History of penile Herpes infection**							
Never	567	97.09	7285	98.50	1		
Ever	17	2.91	111	1.50	1.97	1.17–3.30	0.015
**STIs experience**							
Never	500	86.81	7022	95.06	1		
1 STI	62	10.76	319	4.32	2.73	2.05–3.64	
2 STIs	12	2.08	42	0.57	4.01	2.10–7.67	
3 STIs	2	0.35	4	0.05	7.02	1.28–38.43	<0.001

Among the case group of HIV positive men, 15.5% had a history of having had sex with another man. The HCV seroprevalence was 1.8% in the comparison group of men and 7.2% among HIV positive subjects.

By multivariate analysis, a significant interaction was found between education level and a history of sex with another man. Therefore, we reported the independent effect of risk factors for HIV infection stratified by level of education between those conscripts who had a college education and those who did not. Among those with a college degree, the significant risk factor for HIV infection was a history sex with another man (adjusted OR 23.04, 95% CI 10.23–51.90) after adjusting for age, a history of sex with a FSW and HCV seropositivity. Among those conscripts without a college degree, the factors independently associated with HIV prevalence were HCV seropositivity (adjusted OR 3.89, 95% CI 2.56–5.90), a history of sex with another man (adjusted OR 3.73, 95% CI 2.70–5.13), older age in years (adjusted OR 1.38, 95% CI 1.28–1.48) and a history of sex with a FSW (adjusted OR 1.35, 95% CI 1.10–1.66) (Table 4).

**Table 4:** Mutivariate analysis of the independent risk factors for HIV infection.

Characteristic	With a college degree		Without a college degree	
	Adj. Odds Ratio (95% C.I.)	P-Value	Adj. Odds Ratio (95% C.I.)	P-Value
**Age (years)**	1.04 (0.85–1.23)	0.68	1.38 (1.28–1.48)	<0.001
**History of female sex worker visit**	0.38 (0.14–1.08)	0.07	1.35 (1.10–1.66)	0.005
**History of sexual relation with men**	23.04 (10.23–51.90)	<0.001	3.73 (2.70–5.13)	<0.001
**HCV Ab Positive**	2.60 (0.51–13.39)	0.25	3.89 (2.56–5.90)	<0.001

## Discussion

This study is the largest recent epidemiological study of HIV prevalence and risk factors for HIV infection among a large sample of young adult men in Thailand. These data represent an important re-examination of the current prevalence of HIV among young Thai men and the changes in sexual and other risk behaviors associated with HIV infection after the peak of the HIV epidemic in the 1990s. Recent patterns of risk behaviors among young Thai men have changed substantially from the risk behavior patterns during the peak of the HIV epidemic. We found changing sexual behavioral patterns related to HIV infection among sequential cohorts of RTA conscripts between the early 1990s and 2009 among representative samples of young Thai men. During the early 1990’s soon after the epidemic in IDUs, several reports demonstrated the major role of heterosexual transmission in Thailand especially from having sex with FSWs [3, 15–17]. In 1991, a cross-section survey among 2417 military conscripts in Chiang Mai, Thailand, found that 96.5% of HIV-1 infected men reported a history of sex with a FSW while 1.4%, reported a history of injection drug use [16]. Form the current study, our data confirm the important role of sexual contact between men in the current HIV epidemic in Thailand in addition to the continuing risks of sex with female sex workers and injection drug use. We also demonstrated a re-emergence of the HIV epidemic in the upper-northern and eastern regions of the country similar to the HIV epicenter during the 1990’s [11, 12, 18].

At the national level, we observed that high risk sexual behaviors among young Thai men had been increasing during the recent five-year study period. The increasing high risk sexual behaviors included an increasing number of lifetime sexual partners, and a history of sex between men. Moreover we found a decreasing proportion of men who reported always using a condom with a FSW and a male sex partner during the last 6 months, and a decreasing age at first sexual intercourse.

In addition we found different risk factor patterns among those new conscripts who had a college degree and those who did not. Among those without a college degree, the risk factors associated with HIV infection included age, a history of sex with a FSW, HCV seropositivity, and a history of sex with another man. Among those conscripts who had a college degree, which accounted for 8% of the study population, the only independent risk factor for HIV infection was a history of having had sex with another man. The adjusted odds ratio for a history of having had sex with another man was 3.73 (95% CI 2.70–5.13) among new conscripts without a college degree, while it was 23.04 (95% CI 10.23–51.90) among new conscripts with a college degree. One might hypothesize that Thai men with a lower educational level may have had more limited access to information and resources including access to condoms to prevent HIV infection, have greater access to illicit drugs and more frequent visits to FSWs than those who had higher educational levels. MSM with a college degree may have a higher sexual contact frequency than those with a lower educational level due to the higher accessibility to the internet and other resources to recruit several anonymous partners. This possibility should be evaluated in future research.

Sexual contact with FSWs is still a significant risk factor for HIV infection among young Thai men as it has been since the beginning of the HIV epidemic in Thailand. However, we found a substantially lower and stable proportion of men (23.4–27.3%) reporting a history of sex with a FSW during the recent study period compared with reports of sex with a FSW among 80 to 90% of conscripts when the HIV epidemic emerged in Thailand in the early 1990s [2]. These findings emphasize the importance of sustaining the effective public health interventions, including promoting compliance with 100% condom usage during commercial and casual sex that was very successful in decreasing HIV transmission early in the HIV epidemic in Thailand [2].

Our data suggest that the HIV epidemic has re-emerged in the upper-northern and eastern regions of the country [11, 12, 18]. Another study also reported a higher prevalence of non-gonococcal urethritis among RTA conscripts in the upper northern and eastern regions of the country [19]. More effective public health interventions to prevent HIV infection are especially needed in these high risk regions.

One of the known effective HIV prevention programs that should be emphasized among this young male population is HIV voluntary counseling and testing (VCT). Our study found that 18.9% (95% CI, 17.98–19.85) of young men aged 17 to 29 years reported a history of having been tested for HIV before induction in the military during the five year study period. Thus increasing coverage of the VCT program is an important public health goal in this population.

At the end of 2013, UNAIDS reported an estimate of 35 million (33.2 million–37.2 million) people living with HIV and an annual incidence of 2.1 million people with newly acquired HIV infection worldwide. One third of new HIV infections were among youth (15–24 years old). Thailand had the 19^th^ largest number of people living with HIV [20]. In order to control the current HIV/AIDS epidemic effectively, knowledge about the current epidemic at local and national levels is critically important. According to our findings, the on-going prevention programs in FSWs should be strengthened. Effective interventions specifically designed for MSM both in urban and rural settings of the country should be implemented. The persistent high rate of HIV infection among young MSM is a global problem that has become increasingly important among young men in Thailand [21, 22]. MSM in Thailand constituted about 4% of our study population but the HIV prevalence was about eight-fold greater than among men without such a history. Public health attention has focused on exploring methods to prevent HIV transmission among MSM, such as pre and postexposure prophylaxis with antiretroviral drugs, periodic testing and treating of HIV positive cases and promoting condom and lubricant use during anal sex. The higher HIV seroprevalence among men with a college education is especially concerning.

One of the limitations of the present study was that we were not able to do interviews to evaluate the behavioral risks for HIV in some of the HIV seropositive conscripts. By the time the HIV test results became available at the third month after induction, the basic training for new conscripts had finished, and most of the individuals who were not interviewed had been assigned to their designated units, and therefore were unreachable. Some of the HIV positives were on field maneuvers or had other duties connected to other army units and were unavailable to be interviewed. A small number of IDUs might have left the RTA prior to the interview because they could not tolerate the drug withdrawal symptoms. The low response rate among HIV positives may lead to some selection biases toward the null for some HIV risk factors especially among drug users.

## Conclusions

In conclusion we report epidemiological information on the prevalence and risk factors for HIV among a randomly selected national sample of young Thai men during 2005–2009. Our data suggest that heterosexual sex with FSWs continues to be associated with HIV prevalence among young men in Thailand. Although condom use remains normative for sex with FSWs the rates of consistent condom use has been decreasing recently. The prevalence of HIV among MSM is about eight-fold higher than among men without a history of sex with another man.
